# Microspatial Distributional Patterns of Vectors of Cutaneous Leishmaniasis in Pernambuco, Northeastern Brazil

**DOI:** 10.1155/2012/642910

**Published:** 2012-01-18

**Authors:** Maria Rita Donalisio, A. Townsend Peterson, Pietra Lemos Costa, Fernando José da Silva, Hélio França Valença, Jeffrey J. Shaw, Sinval P. Brandão Filho

**Affiliations:** ^1^Departmento de Medicina Preventiva e Social, Faculdade de Ciências Médicas, UNICAMP, Rua Tessalia Vieira de Camargo 126, 13083-887, Campinas, São Paulo, Brazil; ^2^Biodiversity Institute, University of Kansas, 1345 Jayhawk Boulevard, Lawrence, 66045, USA; ^3^Instituto Aggeu Magalhães, Fundação Oswaldo Cruz, Avenida Moraes Rego s/n, 50670-420 Recife, PE, Brazil; ^4^Instituto de Ciências Biomédicas, Universidade de São Paulo, Avenida Prof. Lineu Prestes 2415, 05508-900 São Paulo, SP, Brazil

## Abstract

The purpose of this study is to analyze the spatial distribution and population trends through time of *Lutzomyia* species in a long-term focus of cutaneous leishmaniasis transmission in an Atlantic Forest area, northeastern Brazil. Sand fly populations of different ecological niches were monitored spatiotemporally in 2009. To summarize vegetation characteristics and phenology, we calculated the Normalized Difference Vegetation Index from Landsat images. Using niche modeling approaches, we assessed suites of environmental factors to identify areas of transmission risk. Although 12 species were detected, *L. whitmani* was the most abundant and broadly distributed across the area, particularly in peridomiciliary locations, and associated negatively with denser vegetation areas. On the other hand, *L. complexa*, *L. sordelli*, and *L. tupynambai* were found almost exclusively in forested areas (*P* < 0.05), and associated positively with denser vegetation. *Lutzomyia* species' occurrences are related to specific environmental combinations (with contrast among species) in the region.

## 1. Introduction

American Cutaneus Leishmaniasis (ACL) is a vector-borne zoonotic disease caused by several species of *Leishmania* trypanosome protozoan parasites and transmitted by phlebotomine sandflies (*Lutzomyia* spp.). In Brazil, ACL constitutes a significant health problem, with an incidence of about 20,153 new cases per year (2008) [1]. In humans, infections may not be readily apparent but can present a variety of clinical manifestations ranging from localised, sometimes self-healing cutaneous lesions to severe mutilating mucocutaneous lesions or diffuse cutaneous leishmaniasis [2]. A high prevalence of infection has been reported in Pernambuco state, northeastern Brazil, concentrated predominantly in the Atlantic Forest region; transmission has increased dramatically in recent decades [3, 4].

Many different patterns of ACL etiology have been described in Brazil, particularly in highly endemic regions [5]. ACL was originally considered to be focused among people who work or live within tropical forests; however, comparison of this idea with current patterns of occurrence suggests strongly that behavior of vectors may be changing, perhaps in response to environmental shifts [1, 6, 7]. Numerous studies point to the capacity of some *Lutzomyia *species to adapt to human-altered environments in parts of Brazil [1, 8–10], which opens possibilities for broader and much increased transmission.

The sandflies *Lutzomyia whitmani* and *L. migonei* are considered important ACL vectors in Brazil that have generally been found in peridomestic environments in southeastern (states of São Paulo, Minas Gerais, Espírito Santo, Rio de Janeiro) [11–13], northeastern (Maranhão, Bahia, Ceará, Pernambuco) [14–16], and west-central (Mato Grosso, Mato Grosso do Sul, Tocantins) regions [10, 17–19]. *Lutzomyia intermedia*, on the other hand, dominates and is considered the principal ACL vector in areas of São Paulo, Rio de Janeiro, and Minas Gerais [1, 11, 12]. Various factors have been identified as key in this domiciliation process, including climatic factors (annual and seasonal temperature and precipitation), vegetation type, and elevation, as well as socioeconomic conditions that may influence risk of transmission to humans [20, 21]. Finally, *Lutzomyia whitmani* ranks among the most important ACL vectors in Brazil [22] and has been found to be abundant in Atlantic Forest areas in Pernambuco since at least the 1990s [4, 16]. This species has been found to be infected naturally with *Leishmania *(*Viannia*) *braziliensis *in this region, forming a key element in the zoonotic transmission cycle of this pathogen [23, 24].

The purpose of this study is to analyze the “behavior” in terms of distribution and population trends through time and across space of *Lutzomyia* species in a long-term focus of ACL transmission in the state of Pernambuco, Brazil. Given that *L. whitmani* dominates the sandfly fauna in this region almost absolutely [16], we focus on this species, as it clearly drives much of the dynamics of the system. We explore this species in relation to various environmental factors in Amarají, Pernambuco, an area of intermingled Atlantic Forest and farmland that is of particular interest as regards high ACL transmission [3, 24].

## 2. Methods

**Figure 1:**
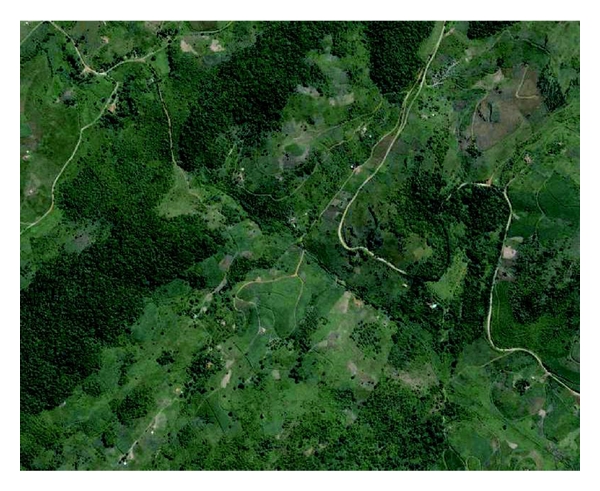
The Amarají landscape Pernambuco, Brazil, 2009, scale = 1 : 24,000.

During the course of 2009, sandflies were collected in the municipality of Amarají, an Atlantic Forest-dominated locality just inland from the coast of Pernambuco. Specifically, we collected at the small, rural settlements of Refrigerio and Tranquilidade (8°22′59′′S 35°27′09′′W, 289 m; Figure 1). The sampling covered 9 months of 2009 (January, February, March, April, June, August, October, November). We used 10 CDC light traps [25] per night on 3-4 nights, for a total of 255 trap-nights over the course of the study. The sampling squeme was habitat based—one CDC light trap inside domiciles, and four in peridomicile (including animal shelters), and five in nearby forested areas. The traps were positioned 1.5 m above the ground on the edges of banana plantations or Atlantic Forest fragments, in the interior of forest fragments, and around human domiciles and associated structures (stables, granaries, etc.). Each site was visited every second month, and the two sets of sites were sampled in alternating sets of months to maximize numbers of sites included in the study. An initial analysis of temporal dimensions sandfly abundance in this study has already been published [16]. The location of each trap was georeferenced using a hand-held Garmin (eTrex HC series) global positioning unit, accurate to ~10 m on the ground using the datum WGS 1984.

The species *Lutzomyia whitmani* was analyzed separately from the other species occurring in the area because of its near-absolute dominance among the sandfly fauna of the region [16]. Species were identified based on morphology and recent keys [26]. We used chi-squared tests to compare relative frequencies of different species in different environments, both in terms of numbers of individuals captured and numbers of sites at which the species were collected. We compared *L. whitmani* with the remaining *Lutzomyia* species except as constrained by sample sizes for the latter species (expected frequencies had to be ≥5 for tests to be possible). All statistical tests were based on an *α* = 0.05.

To summarize dimensions of land cover and vegetation characteristics and phenology, we calculated the Normalized Difference Vegetation Index (NDVI) from Landsat images available from the U.S. Geological Survey (http://glovis.usgs.gov/) with spatial resolution of 30 m (98 ft). Specifically, we used images from the Enhanced Thematic Mapper-7 sensor for the following dates: 3 October and 21 October 2006, 15 April 2007, and 8 August 2009, which were the most cloud-free images available over the period 2006–2009. To calculate NDVI, we used the formula 10,000[(B40 − B30)/(B40 + B30)], where B40 and B30 represent the red and near-infrared bands from the Landsat images, respectively.

To identify environmental conditions under which *Lutzomyia *species occur in the Amarají region, we used ecological niche modeling (ENM) routines implemented in Maxent [27]. In general, we used default parameters to train models, so that we set the random testing percentage to 50% to provide an independent perspective on model quality; we focused on the logistic output format. To identify environmental parameters most relevant to the species' occurrence, we used a jackknife procedure that measures effects of each environment variable alone and when omitted from the model on predictions [27, 28].

To separate areas predicted as suitable from those predicted as unsuitable [29], we set the expected meaningful error parameter of Peterson et al. (2008) [30] as *E* = 2%. We used as a threshold for separating prediction of suitability from prediction of unsuitability the highest logistic Maxent suitability value that included (100−*E*)%, in this case 98% of the training data, to take into account the possible presence of noise in the input data.

To provide a quantitative test of model predictions, we were forced to focus on a subset of the study area, for which no cloud cover was present in at least two of the images (Figure 1). To provide a clear test of predictive ability among spatial subsets, thereby avoiding some problems with spatial autocorrelation among training and testing data, we separated this subset of the study region into eastern, central, and western areas and challenged models to use each pair of areas to anticipate the distribution of the species in the third area. We then used a receiving operating characteristic (ROC) approach to provide a threshold-independent evaluation of each prediction [30]. However, in light of known problems with standard ROC approaches [31, 32], we used a partial ROC approach, in which analyses are limited to portions of the ROC curve in which omission error is less than or equal to *E*; these tests were developed using a program developed by N. Barve that is available upon request from the authors. The test statistic output from these routines is the AUC ratio, which compares the observed area under the curve to null expectations; AUC ratios are tested for difference from unity (i.e., random prediction) via 1000 repetitions of a 50% bootstrap of available input data.

Finally, to provide a visualization of ecological niche patterns of *Lutzomyia* species' distributions in the study area, we developed a final niche model based on all occurrence data available across the three areas (i.e., no subsetting). We combined (grid COMBINE in ArcGIS 9.3) this map with the environmental data layers from which it was derived to yield a raster dataset with an associated attributes table that includes the value of each combination of environmental variables (NDVI of each time period) and the prediction from Maxent. This table was exported in ASCII format and used to develop various visualizations of niche patterns.

## 3. Results

We identified 12 *Lutzomyia* species in the Amarají study during 2009, for a total of 1361 individuals across the 255 trap sites. *Lutzomyia whitmani* was by far the most abundant species, totaling 1195 individuals (87.8%; Table 1). Other species recorded included *L. evandroi* (4.9%), *L. quinquefer* (1.9%), and *L. complexa* (1.3%), among others. 

In peridomiciliary environments, *L. whitmani* was even more dominant (98.2% of individuals); *L. evandroi*,* L. quinquefer*, and *L. migonei* appeared to show a similar association with human-modified environments, albeit in much lower numbers. In contrast, *L. complexa*, *L. tupynambai*, *L. sordelli*, *L. longispina*, and *L. walkeri* appeared restricted to forest and forest edge in this study (Table 1). The concentration of *L. whitmani* in peridomiciliary environments was much greater than would be expected by chance (*P* < 0.05); tests for the remaining species were equivocal, probably owing to small sample sizes.

Table 1 shows the distribution of positive sites and individuals species in forested and peridomiciliary areas. Among the 120 sandfly positive sites, 82 (68.3%) were in peridomiciliary areas, while 38 (31.7%) were detected in forested areas (*P* = 0.005). *L. whitmani* and *L. quinquefer* were dominant in peridomestic sites (*P* < 0.05), as well as *L. migonei* (*P* = 0.07), while *L. complexa, L. sordelli*, and *L. tupynambai* were found almost exclusively in forested areas (*P* < 0.05). For all other species, distribution of positive sites among peridomiciliary versus forested areas could not be determined (*P* ≫ 0.05). *L. whitmani* was the most abundant species as well as the most broadly distributed across the study area.

Relating patterns of occurrence to patterns of surface reflectance in the Landsat imagery, the jackknife process indicated NDVI from October and April as the environmental variables most associated with presence of *L. whitmani*. NDVI of March and August were omitted from models in light of their little contribution to fitness of models.

Niche models estimated from and projected among the three regions of the study area for which cloud-free imagery was available showed good (i.e., better than random expectances) coincidence with independent testing data sets (Table 2). In particular, for example, for *L. whitmani*, the model based on western and central regions predicted the distribution of the species in the eastern region with an AUC ratio mean of 1.12, which has an associated probability value of *P* < 0.001. The other two predictions were similarly statistically significantly better than random expectations (Table 2) thus amply confirming both the environmental influences on sandfly distribution and the predictive power of our models.

**Figure 2:**
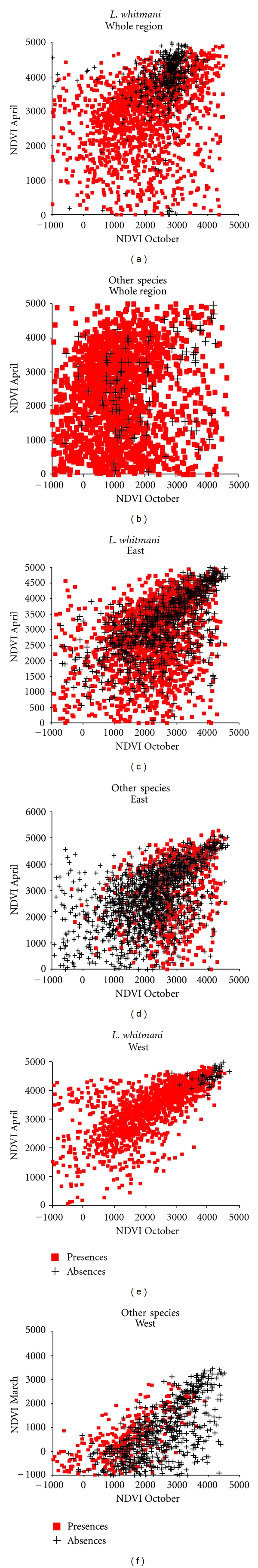
Presences and absences of *Lutzomyia whitmani* (a, c, e) and other species of *Lutzomyia* (b, d, f) in west, east, and center regions of the Amarají study area in Pernambuco, according to NDVI values October 2006 and March 2006.

**Figure 3:**
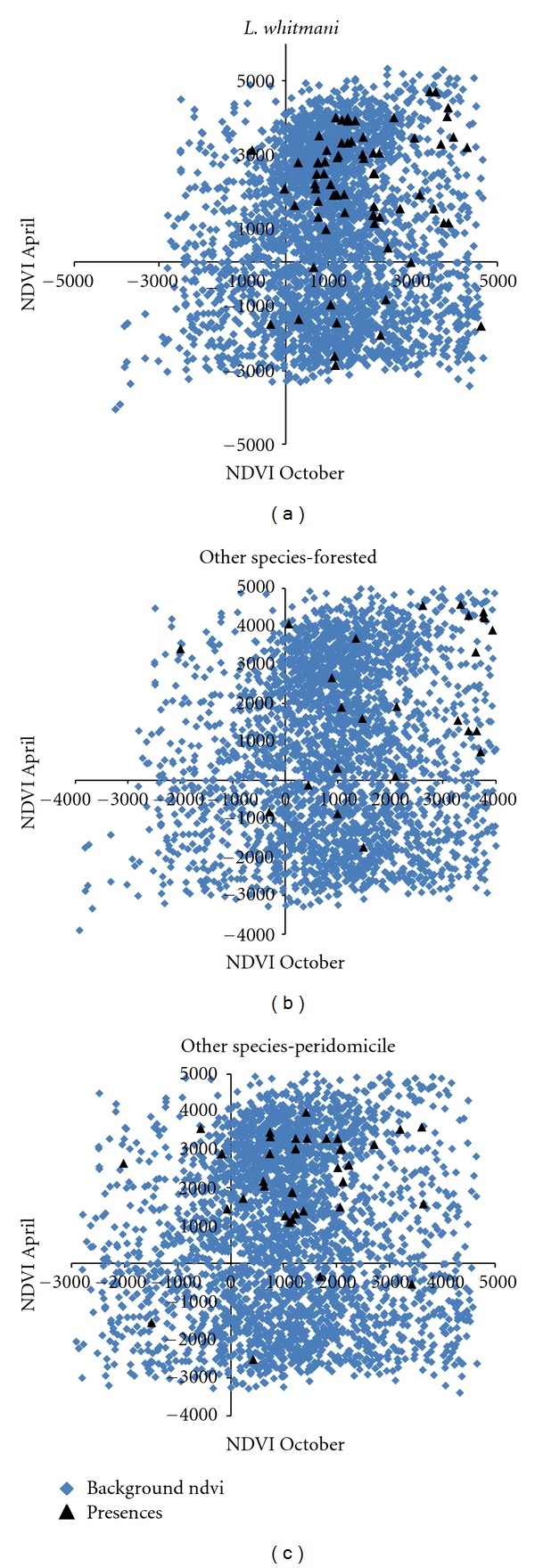
Summary of environmental characteristics of known occurrences (i.e., traps positive) for *Lutzomyia whitmani *versus two sets of other species (one of forested sites, one of peridomiciliary sites; see text for explanation). Occurrences shown as triangles are depicted against the background of conditions available across the study area (in gray). Environmental conditions of NDVI of October and April, Amarají, Pernambuco, Brazil, 2009.

Figure 2 shows the relationship between modeled distributions in environmental space of *L. whitmani *as opposed to the other sandfly species at Amarají, revealing differences in the distribution of species according to the vegetation index.  Figure 3 shows adding detail regarding presences detected for *L. whitmani* versus other species in forested and peridomiciliary environments. *L. whitmani* appears to be associated negatively with NDVI values (both in April, which is the rainy season, and in October, which is the dry season), as areas predicted as unsuitable for this species show generally higher NDVI values (Figures 2 and 3). Most other *Lutzomyia* species, on the other hand, appear associated positively with denser vegetation (i.e., higher NDVI values) in both seasons (Figures 3). Overall, then, *Lutzomyia* species' occurrences are associated with specific environmental combinations (with contrast among species) in Amarají.

## 4. Discussion 

In the Amarají region as well as in other areas of Brazil, *Lutzomyia whitmani* has been implicated as the principal ACL vector, predominantly associated with *Leishmania braziliensis,* the main parasite species involved in transmission, although in southern region of the country it is considered only a secondary vector [3, 13]. In Amarají, although 12 species were detected, *L. whitmani* was dominant constituting 87.8% overall of detections, and 95% in peridomiciliary locations. In forested areas, *L. whitmani* was less dominant (only 41.7% of detections), and other species played more meaningful roles in the sandfly community. This tie of *L. whitmani *to human-altered environments has been noted also in Amazon Basin, and in the center-west and southern parts of the country [10, 11, 14].

However, in a nearby region of Pernambuco with greater forest cover, *L. whitmani* was found to be a relatively unimportant member of the sandfly community, and *L. complexa* and *L. choti* were much more numerous [33]. Among the other species detected at Amarají, *L. evandroi *and *L. migonei *both also appeared to be concentrated in peridomiciliary environments. Although far less common than *L. whitmani*, the human association of these species makes them of some interest in ACL transmission, as in other regions [11, 34, 35].

Given its domiciliation and massive dominance, *L. whitmani* is almost certainly the major ACL vector in the region [24, 36]. While other species were rare around human habitations, this sand fly was more abundant in peridomiciliary and to a lesser degree forested areas, offering a possible vectorial role for other *Leishmania* species in the wooded environments. Detection of *L. whitmani* in peridomestic and forested areas reinforces the assumption that deforestation does not result in decline of the species habitat but adaptation and/or tolerance of different vegetation type and climatic condition.

Clearly, the next step in this process would be detailed analysis of (1) blood meal sources for each sandfly species in the region, (2) detection of *Leishmania* infections in the flies, and (3) identification and association of *Leishmania* strains in both sandflies and locally infected humans. This group of information, together with our spatial data, would offer significant insight into the details of ACL transmission cycle in the region.

Predictive models relating species occurrences to abiotic variables have been used in several previous studies of distribution and ecology of vectors, reservoirs, and infectious diseases [10, 21, 37]. Most previous analyzes have been carried out at scales that are set by resolution of the available environmental variables of occurrence data [21, 38, 39]. However, climate of the relatively small area designated in this study does not vary much over scales like this. Whereas the analysis of existing vegetation indirectly reflects the effects of rain and vegetation in the region, identifying ecologically disturbed and forested areas is of particular interest in the study of *Lutzomyia* spp. The amount of vegetation in the dry and wet seasons were adequate in predictions of species occurrence, particularly for *L. whitmani,* which could be analyzed in detail. For this purpose, NDVI provides a good index of ecosystem function with strong correlation with absorbed photosynthetically active radiation [39].

In spite the fact that *L. whitmani* has been associated with geoecological factors, this highly anthropophilic species is also influenced by socioenvironmental changes and transformation on landscape [10, 34], which were not evaluated in this study.

There was a negative association between this species and higher NDVI values (denser vegetation), and predictions of the distribution of *L. whitmani* among regsions were statistically significantly better than random expectations. This result strongly suggests that it is feasible to predict the distribution of this important vector in regions where it is difficult to perform sampling due to factors such as difficult access and financial restrictions.

## Figures and Tables

**Table 1:** Frequency of positive sites and individuals of species of *Lutzomyia* sandflies identified in forested and peridomiciliary areas in Amarají, Permambuco, Brazil, in 2009. More than one species of *Lutzomyia* was found at some sites.

Species	Sites (*n* = 255)			Individuals (*n* = 1,361)			Total individuals
	Peridomicile forested			Peridomicile forested			
	(*n* = 130)	(*n* = 125)	*P*	Freq (%)	Freq (%)	*P*	Freq (%)
	Freq (%)	Freq (%)					
*L. whitmani*	69 (82.1)	15 (17.9)	<0.001	1135 (95.0)	60 (5.0)	<0.001	1195 (87.8)
Other species:	44 (57.9)	32 (42.1)	0.41	82 (49.4)	84 (50.6)	1	166 (12.2)
*L. evandroi*	22 (66.7)	11 (33.3)	0.18	43 (64.2)	24 (35.8)	0.10	67 (4.9)
*L. quinquefer*	17 (89.5)	2 (10.5)	0.015	23 (88.5)	3 (11.5)	0.006	26 (1.9)
*L. complexa*	1 (10.0)	9 (90)	0.07	1 (5.6)	17 (94.4)	0.007	18 (1.3)
*L. sordelli*	0 (0)	8 (100)	—	0 (0)	16 (100)	0.005	16 (1.2)
*L. tupynambai*	0 (0)	2 (100)	—	0 (0)	15 (100)	0.006	15 (1.1)
*L. migonei*	9 (90.0)	1 (10)	—	10 (90.9)	1 (9.1)	0.06	11 (0.8)
*L. fischeri*	2 (66.7)	1 (33.3)	—	2 (66.7)	1 (33.3)	—	3 (0.2)
*L. capixaba*	1 (33.3)	2 (66.7)	—	1 (33.3)	2 (66.7)	—	3 (0.2)
*L. walkeri*	0 (0)	3 (100)	—	0 (0)	3 (100)	—	3 (0.2)
*L. naftalekatzi*	1 (100)	0 (0)	—	2 (100)	0 (0)	—	2 (0.1)
*L. longispina*	0 (0)	2 (100)	—	0 (0)	2 (100)	—	2 (0.1)

Total negative (135)	48 (35.6)	87 (64.4)	0.02				
Total positive (120)	82 (68.3)	38 (31.7)	0.005	1217 (89.4)	144 (10.6)	<0.001	1361 (100)

**Table 2:** Areas predicted as suitable for *Lutzomyia whitmani* by ecological niche model based on NDVI variables for combination of the east, west, and center regions of Amarají, Pernambuco, Brazil.

	Regions	Ratio AUC*	Variables associated	Partial ROC N° ratio ≤ 1 out of 1000	*Z* statistics (*P*)
*L. whitmani*	East and Center predict *West *	1.06	NDVI October	0	<0.0001
	West and Center predict *East *	1.12	NDVI April	0	<0.0001
	West and East predict* Center *	1.10	NDVI October	0	0.03
	Whole region of Amarají		NDVI March		—

*Area under the ROC curve, expressed as a ratio of observed AUC to the area under the curve of random expectations.
